# Association between Central Serous Chorioretinopathy and Risk of Depression: A Population-Based Cohort Study

**DOI:** 10.1155/2019/2749296

**Published:** 2019-05-02

**Authors:** Yu-Yen Chen, Li-Ying Huang, Wei-Ling Liao, Pesus Chou

**Affiliations:** ^1^Department of Ophthalmology, Taichung Veterans General Hospital, Taichung 407, Taiwan; ^2^School of Medicine, National Yang-Ming University, Taipei 112, Taiwan; ^3^School of Medicine, College of Medicine, Fu Jen Catholic University, New Taipei 242, Taiwan; ^4^Division of Endocrinology and Metabolism, Department of Internal Medicine, Fu Jen Catholic University Hospital, New Taipei 243, Taiwan; ^5^Department of Medical Education, Fu Jen Catholic University Hospital, New Taipei 243, Taiwan; ^6^Nurse Department of Taichung Veterans General Hospital, Taichung 407, Taiwan; ^7^Community Medicine Research Center and Institute of Public Health, National Yang-Ming University, Taipei 112, Taiwan

## Abstract

**Purpose:**

To investigate the association between central serous chorioretinopathy (CSC) and the risk of developing depression. The risk factors associated with depression in CSC patients were also assessed.

**Methods:**

A population-based retrospective cohort study using the Taiwan National Health Insurance Research Database was conducted from the beginning of 2001 through the end of 2013. CSC patients and age- and gender-matched (1 : 4 matched) control subjects without CSC were enrolled in the study. Kaplan–Meier curves were generated to compare the cumulative hazard of subsequent depression between the CSC and control groups. A Cox regression analysis estimated the crude and adjusted hazard ratios (HRs) for depression. Risk factors leading to depression were investigated among the CSC patients.

**Results:**

25,939 CSC patients and 103,756 controls were enrolled in the study. The CSC group had a significantly higher cumulative hazard for depression compared to the control group (*p* value < 0.0001). The Cox regression model indicated that the CSC group had a significantly higher risk for depression (adjusted HR = 1.33). Within the CSC group, significant risk factors for depression included age, female gender, low income, first-onset CSC, peptic ulcer, and smoking. The recent use of steroids prior to CSC, by all routes of administration, also significantly increased the risk for depression. However, treatment of CSC did not significantly reduce the risk for depression.

**Conclusion:**

Patients with CSC are at significantly greater risk of developing depression. Among CSC patients, age, female gender, low income, first-onset CSC, peptic ulcer, smoking, and recent use of steroids prior to CSC were significant risk factors for depression.

## 1. Introduction

Central serous chorioretinopathy (CSC) is characterized by serous neurosensory retinal detachment of the macula with circulatory disturbances of the choroidal vasculature [1]. The symptoms of CSC include micropsia, metamorphopsia, reduced color sensitivity, and acute/subacute central vision loss or distortion [2]. Most cases of CSC are self-limiting with spontaneous resolution. However, recurrences are common [3, 4]. Although the etiologies of CSC remain unclear, many precipitating factors have been found, including stress, type A personality traits, and steroid use [1, 5–7].

The psychological stress and type A personality traits that are risk factors for CSC are also risk factors for depression [8, 9]. In addition, CSC and depression have another common risk factor—steroid use [10]. Furthermore, visual symptoms in CSC may increase the risk of depression. Taken together, it is reasonable to speculate that there may be a relationship between CSC and depression.

However, there are no previous studies investigating the association between CSC and subsequent depression. This may be due to the insufficient number of hospital-based cases required to conduct this kind of study. In addition, many variables, such as age, gender, comorbidities, and economic status, also have an impact on the development of depression and should be regarded as confounders. Therefore, in our study, we utilized the whole population database in Taiwan to investigate whether CSC patients have a higher risk of subsequent depression. In addition, risk factors associated with the development of depression among CSC patients were explored. Because of the completeness of the database, we can adjust the effect of confounders and derive convincing results.

## 2. Materials and Methods

### 2.1. Study Setting

This study was approved by the Ethical Committee of Yang-Ming University Hospital (2015A017). We utilized the National Health Insurance Research Database (NHIRD), which includes the registry files and healthcare records of over 99% of Taiwan's 23 million residents. NHIRD is maintained by the National Health Research Institutes of Taiwan, and the data are released for research purposes after deidentification of each patient. Written informed consent was exempt according to the rules of the Institutional Review Board. A large number of studies have been conducted utilizing the database, including our previous works [11, 12]. This study is a novel study with a different topic. We sought to compare the risk of depression in subjects with and without CSC during a 13-year period.

### 2.2. Inclusion and Exclusion Criteria

Using the Taiwan NHIRD from 1996 to 2013, we performed a retrospective cohort study. Patients with a diagnosis of CSC from 2001 to 2013 according to the International Classification of Diseases, 9th Revision, Clinical Modification Codes (ICD-9-CM code: 362.42) were selected for the CSC group. Patients with a CSC diagnosis from January 1, 1996, to December 31, 2000, were excluded to eliminate patients with previous chronic CSC. The date of the first CSC claim was defined as the index date. We also randomly selected individuals who had never received a diagnosis of CSC as a control group at a ratio of 1 : 4 and matched the control group with the CSC group on age, gender, and index year (the year of index date or enrollment). The two groups were followed until the end of 2013 to see whether they subsequently developed depression (ICD-9-CM codes: 296.2X, 296.3X, 3004, 311). Patients with depression were diagnosed by psychiatrists with well-established diagnostic criteria and protocols. CSC subjects and controls who were diagnosed with depression or a severe mental health disorder prior to the index year were excluded from the analysis.

### 2.3. Study Variables

The covariates included in our statistical analyses were age, gender, and other risk factors for depression (e.g., economic status and severity of systemic diseases). We used the cost of insurance and urbanization level of residency to represent the economic status. The reason is, in our National Health Insurance program, insurance cost is correlated with income. If a patient has a greater income, he/she has to pay more for insurance. In addition, the urbanization level of residency was also an index for economic status. The severity of systemic diseases was measured by Charlson comorbidity index (CCI) [13]. Furthermore, hypertension [14], peptic ulcer [14–16], and smoking (or chronic obstructive pulmonary disease) [16] had to be adjusted in the statistical analyses because they were associated with CSC.

### 2.4. Statistical Analysis

Group differences between the CSC group and the control group were analyzed in age, gender, CCI, insurance cost, urbanization level of residency, hypertension, peptic ulcer, and smoking. The two groups were compared using two-sample *t*-tests for continuous variables and chi-squared tests for categorical variables. We applied a survival analysis using the Kaplan–Meier estimator with a log-rank test to describe and compare the cumulative hazard curves for depression in the two groups. The univariate and multivariate Cox proportional hazard models were used to estimate the hazard ratio (HR) for the occurrence of depression according to each variable. The variables included in the regression analysis were CSC, age, gender, CCI, insurance cost, urbanization level of residency, hypertension, peptic ulcer, and smoking.

Subsequently, we focused on the CSC patients and applied Cox regression to investigate the risk factors associated with depression among the CSC patients. The variables included in the analysis were age, gender, CCI, insurance cost, urbanization level of residency, hypertension, peptic ulcer, smoking, recurrence of CSC, and types of steroid medication. Prior use of steroid medications was identified and classified by the National Drug Code and the Anatomic Therapeutic Chemical (ATC) code. According to the classification system, steroid medications include systemic (intravenous/oral), inhaled/nasal, cutaneous, and topical (eye drops/ointments). Users were defined as those who had used medications in the last 6 months before the index date. Finally, the CSC patients were classified into subgroups according to treatment or not. If a CSC patient received treatment such as focal laser, photodynamic therapy with verteporfin, intravitreal injection of antivascular endothelial growth factor agents, or oral mineralocorticoid antagonists, he or she was classified into the “with treatment” subgroup. If a CSC patient never received any treatment, he or she was classified into the “without treatment” subgroup. Then, the incidence density of depression was compared between the two subgroups. The adjusted HR for depression was calculated to derive the impact of treatment on the risk of depression after adjustment of confounders. All statistical operations were performed using the SAS statistical package, version 9.2 (SAS Institute, Cary, NC, USA).

## 3. Results

### 3.1. Comparison of the Demographic and Clinical Characteristics between the Two Groups

In the study, 25,939 CSC patients and 103,756 matched controls were enrolled.  Table 1 displays the demographics and clinical characteristics of the two groups. The mean age in both groups was 42.9 years. The CSC group had significantly higher insurance costs than the control group. The CSC group also had significantly higher prevalences in hypertension, peptic ulcer, and smoking. During the 13-year study period, the CSC group had a significantly higher cumulative incidence of depression (6.0%) than the control group (4.6%).

### 3.2. Cumulative Hazard Curves by the Kaplan–Meier Method

**Figure 1:**
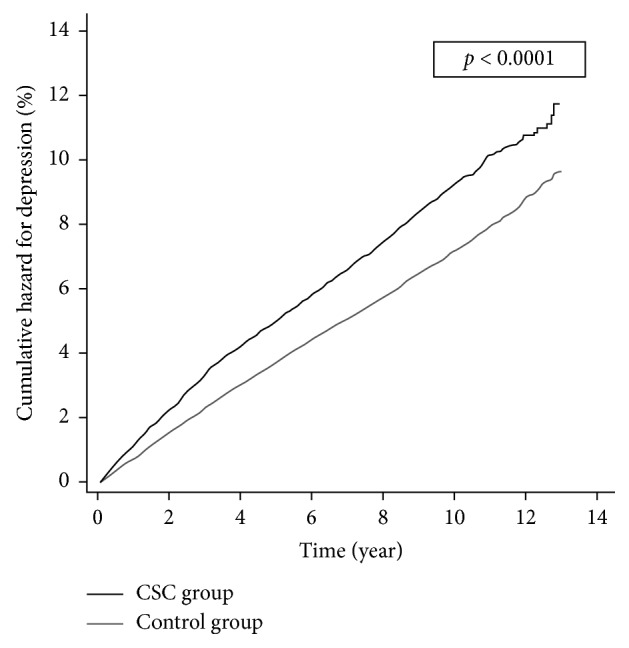
Kaplan–Meier curves for depression among CSC patients and the control group. The black line represents the CSC group, and the gray line represents the control group.

Figure 1 illustrates the cumulative hazard curves for depression in the CSC group and the control group. The Kaplan–Meier method with a log-rank test revealed a statistically significant difference between the hazard curves of the two groups (*p* value <0.0001).

### 3.3. Univariate and Multivariate Analyses by the Cox Regression Model

Table 2 shows the unadjusted HR for depression was 1.32 times greater in the CSC group compared to the control group (95% confidence interval (CI): 1.25–1.40). The significantly greater hazard for depression in the CSC group remained after adjusting for confounders (adjusted HR = 1.29; 95% CI: 1.22–1.34). Besides, age was a significant risk factor for depression in both univariate and multivariate analyses. The adjusted HR for depression in individuals over 70 years old was 1.61 times greater when compared with those less than 40 years old. Males were less likely to develop depression than females (adjusted HR = 0.68; 95% CI: 0.65–0.72). The risk of developing depression was significantly higher in patients with a greater CCI (adjusted HR = 1.17; 95% CI: 1.08–1.27). However, patients with a higher insurance cost had a significantly lower risk of depression (adjusted HR = 0.76; 95% CI: 0.72–0.81). Residential urbanization was not significantly related to the risk of depression in both univariate and multivariate analyses. As far as hypertension was concerned, it was not significantly associated with depression in the multivariate analysis. Nevertheless, peptic ulcer and smoking both significantly increased the risk of depression.

### 3.4. Risk Factors for Depression among CSC Patients

Table 3 reveals the risk factors for depression focusing on the CSC patients. Older age, female gender, first-onset CSC, and a lower insurance cost significantly increased the risk of developing depression among CSC patients in univariate as well as multivariate Cox regression analyses. CCI was a significant risk factor for depression in the univariate analysis, but the significance did not remain in the multivariate analysis. Residential urbanization was not a significant risk factor for depression in both univariate and multivariate analyses. Regarding steroid use before the diagnosis of CSC, all types of steroids (systemic, cutaneous, local injection, nasal/inhaled, and eye drops/ointments) significantly increased the risk of depression. CSC patients with hypertension did not have a significantly higher risk of depression compared to those without hypertension. However, both peptic ulcer and smoking were significant risk factors for depression among CSC patients.

### 3.5. Stratified Analyses according to Treatment among CSR Patients

In Table 4, the CSC patients were divided into the two subgroups (treatment vs. without treatment). The incidence density of depression was 100.06 and 86.27 per 10000 year in the “with treatment” and “without treatment” subgroups, respectively. CSC patients receiving treatment had a lower risk of developing depression than those without treatment. However, the extent of reduced risk did not reach a statistical significance (unadjusted HR = 0.86 with 95% CI 0.68–1.10; adjusted HR = 0.85 with 95% CI 0.67–1.08).

## 4. Discussion

We conducted a 13-year follow-up study on population-based data from the Taiwan NHIRD. Compared to those without CSC, patients with CSC had a significantly higher risk (HR = 1.29) of developing depression. Among CSC patients, older age, female gender, first-onset CSC, lower income, peptic ulcer, and smoking were significant risk factors for developing depression. Recent use of any type of steroid prior to the diagnosis of CSC also significantly increased the risk of depression among CSC patients.

Our study analyzed the characteristics of CSC patients. Most of them were male, and almost 75% of them were under 50 years old (Table 1). Previous studies also found CSC typically occurred in males in their 20 s to 50 s [17–19]. Our study further revealed that CSC patients had a significantly higher cumulative incidence and cumulative hazard for depression (Table 1 and Figure 1). To the best of our knowledge, previous studies regarding the association between CSC and psychological problems have never investigated the risk of subsequent depression after CSC occurrence. Some studies found that type A personality traits, heightened emotional instability, and insecurity may play a role in CSC [5, 20–25]. Some studies used questionnaires to assess psychological symptoms and found that CSC patients scored significantly higher on depression symptoms than the healthy controls [25, 26]. However, based on their cross-sectional or case-control study design, we cannot determine whether CSC or depression occurs first. Our study, with the strength of cohort study design, can have a clearer time sequence and thus can derive the association between CSC and subsequent depression.

As shown in Table 2, CSC significantly increases the risk of subsequent depression compared with the control group in the univariate Cox regression analysis (unadjusted HR = 1.32; 95% CI: 1.25–1.40). Confounders should be adjusted to derive an accurate association between CSC and depression. In multivariate analysis after adjustment for confounders, CSC is still a significant risk factor for depression (adjusted HR = 1.29; 95% CI: 1.22–1.34). One strength of our study is the completeness and large number of cases from the NHIRD. Demographic data and all the diagnoses as well as prescriptions were accurately recorded in the database. Large case numbers provide sufficient statistical power and enable confounders to be adjusted. In addition, the diagnoses in the database were based on standard diagnostic criteria and thorough examinations. Our National Health Administration (NHA) routinely checks the medical charts to see whether the patients received proper diagnosis and confirms the compatibility between medical charts and claimed data. For example, CSC patients in our study should have been confirmed by findings of fundoscopy, fluorescein angiography, and/or optical coherence tomography. Depression should be diagnosed by board-certified psychiatrists using well-established diagnostic criteria. All the diagnoses in our study were based on the generally accepted ICD9-CM codes, not only from symptoms reported on questionnaires as previous studies.

The explanations that patients with CSC have a higher risk of developing depression are still unclear. Previous studies revealed that CSC in itself, or the visually annoying symptoms, may be associated with stress [21, 23, 24]. Stress induces cortisol, which precipitates depression [8, 27]. In addition, the psychological and personality traits in patients with CSC also create a vulnerability toward depression [28].

Another strength of our study is that we further focused on CSC patients to investigate factors predisposing them to depression (Table 3). To the best of our knowledge, this is the first study to investigate this issue. As in the general population, age and female gender are risk factors for depression among CSC patients. It is noteworthy that the recent use of steroids prior to CSC, regardless of the type of administrative route, significantly increased the risk of developing depression. A possible explanation may be that the cortisol level is associated with depression [27]. Glucocorticoids, endogenous or exogenous, can cause excitotoxicity to pyramidal neurons in the hippocampus, leading to inhibition of neurogenesis in the hippocampus [29]. Glucocorticoids also reduce the volume of hippocampus, thus affecting the function of brain areas related to emotion and reward circuitry, resulting in depression [30]. Even usage of low and medium doses of corticosteroids can induce depression [31]. It is compatible with our finding that even steroid eye drops/ointments or nasal/inhaled steroids may increase the risk of depression.

Another finding of our study is that patients with recurrent CSC were less likely to become depressed than those with first-onset CSC. To the best of our knowledge, our study is the first to address this issue. The explanation is still unknown. The adaptation to visual symptoms in recurrent CSC patients may be the reason for less depression. In addition, recurrent cases have experienced previous CSC episodes and realized the self-limited characteristic of CSC, thus reducing psychological stress. Further studies will be necessary to investigate this issue. We also found that peptic ulcer and smoking were significantly associated with depression. These findings were compatible with previous studies [32, 33].

The limitation of our study is that some individuals with CSC may not go to see a doctor and therefore are not diagnosed with CSC. In our database, they will be classified into the control group, not the CSC group. This misclassification will induce a bias toward the null. Thus, if the risk of developing depression is statistically significant in our analysis, the significance will be more prominent in the real situation. Therefore, the association between CSC and depression is a real phenomenon.

The findings from our study have clinical and public health implications. Clinically, when treating CSC patients, ophthalmologists need to focus not only on the medical aspects of CSC but also on providing psychological support to their patients. CSC patients at a high risk for depression, such as older patients, female patients, patients with peptic ulcer, smoking, and those with first-onset CSC, should be referred to a psychiatrist if early signs of depression become apparent. From a public health perspective, policy makers are encouraged to enforce screening for depression risk in patients with CSC and to provide more substantial and integrated care.

## Figures and Tables

**Table 1:** Characteristics of the study subjects.

Variable	CSC group, *n* = 25939	Control group, *n* = 103756	*p* value
	*n* (%)	*n* (%)	
Age, year (mean ± SD)	42.9 ± 12.3	42.9 ± 12.3	1.000
Age, categorical			1.000
** **<40	10813 (41.7)	43252 (41.7)	
** **40–50	8386 (32.3)	33544 (32.3)	
** **50–60	4163 (16.0)	16652 (16.0)	
** **60–70	1677 (6.5)	6708 (6.5)	
** **≥70	900 (3.5)	3600 (3.5)	
Gender			1.000
** **Male	17751 (68.4)	71004 (68.4)	
** **Female	8188 (31.6)	32752 (31.6)	
Charlson comorbidity index			0.06
** **<2	23265 (89.7)	93470 (90.1)	
** **≥2	2674 (10.3)	10286 (9.9)	
Insurance cost			<0.0001
** **<30000 NTD	17034 (65.7)	71364 (68.8)	
** **≥30000 NTD	8905 (34.3)	32392 (31.2)	
Urbanization level			0.27
** **Urban	5297 (20.4)	21511 (20.7)	
** **Rural	20642 (79.6)	82245 (79.3)	
Hypertension			<0.0001
** **Yes	7626 (29.4)	11711 (11.3)	
** **No	18313 (70.6)	92045 (88.7)	
Peptic ulcer			<0.0001
** **Yes	5508 (21,2)	3277 (3.2)	
** **No	20431 (78.8)	100479 (96.8)	
Smoking			<0.0001
** **Yes	4699 (18.9)	4259 (4,1)	
** **No	21040 (81.1)	99497 (95.9)	
Depression during the follow-up period	1561 (6.0)	4736 (4.6)	<0.0001

SD indicates standard deviation, and NTD indicates New Taiwan Dollar.

**Table 2:** Analyses of risk factors for depression in patients with and without CSC.

Predictive variables	Univariate analysis		Multivariate analysis	
	Unadjusted HR (95% CI)	*p* value	Adjusted HR (95% CI)	*p* value
CSC (yes vs. no)	1.32 (1.25–1.40)	<0.0001	1.29 (1.22–1.34)	<0.0001
Age				
** **30–40	Reference		Reference	
** **40–50	1.04 (0.98–1.10)	0.24	1.08 (1.02–1.15)	0.01
** **50–60	1.30 (1.21–1.39)	<0.0001	1.23 (1.14–1.33)	<0.0001
** **60–70	1.61 (1.46–1.77)	<0.0001	1.33 (1.20–1.47)	<0.0001
** **≥70	2.17 (1.94–2.43)	<0.0001	1.61 (1.42–1.83)	<0.0001
Gender (male vs. female)	0.66 (0.62–0.69)	<0.0001	0.68 (0.65–0.72)	<0.0001
Charlson comorbidity index				
** **<2	Reference		Reference	
** **≥2	1.49 (1.39–1.60)	<0.0001	1.17 (1.08–1.27)	0.0001
Insurance cost				
** **<30000 NTD	Reference		Reference	
** **≥30000 NTD	0.67 (0.64–0.71)	<0.0001	0.76 (0.72–0.81)	<0.0001
Urbanization level				
** **Urban	Reference		Reference	
** **Rural	0.94 (0.86–1.04)	0.25	0.98 (0.93–1.04)	0.55
Hypertension (yes vs. no)	1.40 (1.31–1.48)	<0.0001	1.01 (0.94–1.09)	0.78
Peptic ulcer (yes vs. no)	1.90 (1.76–2.04)	<0.0001	1.53 (1.41–1.66)	<0.0001
Smoking (yes vs. no)	1.66 (1.54–1.79)	<0.0001	1.20 (1.10–1.31)	<0.0001

CSC indicates central serous chorioretinopathy, NTD indicates New Taiwan Dollar, HR indicates hazard ratio, and CI indicates confidence interval. In the multivariable analysis, all the other variables in the table are included for adjustment.

**Table 3:** Analyses of the risk factors for depression among patients with CSC.

Predictive variables	Univariate analysis		Multivariable analysis	
	Unadjusted HR (95% CI)	*p* value	Adjusted HR (95% CI)	*p* value
Age				
** **≤40	Reference		Reference	
** **40–50	1.09 (0.96–1.22)	0.18	1.01 (0.89–1.14)	0.90
** **50–60	1.27 (1.19–1.47)	0.001	1.05 (0.90–1.24)	0.52
** **60–70	1.59 (1.32–1.93)	<0.05	1.18 (0.96–1.46)	0.13
** **≥70	1.56 (1.22–1.99)	<0.0001	1.59 (1.23–2.04)	0.0004
Gender (male vs. female)	0.68 (0.62–0.75)	<0.0001	0.69 (0.62–0.76)	<0.0001
State of CSC				
** **First onset	Reference		Reference	
** **Recurrence	0.50 (0.45–0.56)	<0.0001	0.45 (0.40–0.50)	<0.0001
Charlson comorbidity index				
** **<2	Reference		Reference	
** **≥2	1.40 (1.25–1.58)	<0.0001	1.04 (0.91–1.18)	0.60
Insurance cost				
** **<30000 NTD	Reference		Reference	
** **≥30000 NTD	0.69 (0.61–0.77)	<0.0001	0.80 (0.72–0.90)	0.0002
Urbanization level				
** **Urban	Reference		Reference	
** **Rural	1.10 (0.99–1.22)	0.06	1.08 (0.97–1.19)	0.15
Types of steroid medications				
** **Systemic (yes vs. no)	1.55 (1.38–1.74)	<0.0001	1.21 (1.08–1.36)	0.002
** **Cutaneous (yes vs. no)	1.96 (1.70–2.25)	<0.0001	1.64 (1.41–1.90)	<0.0001
** **Local injection (yes vs. no)	1.68 (1.52–1.86)	<0.0001	1.38 (1.24–1.54)	<0.0001
** **Nasal/inhaled (yes vs. no)	1.67 (1.48–1.87)	<0.0001	1.47 (1.30–1.67)	<0.0001
** **Eye drops/ointments (yes vs. no)	1.40 (1.22–1.61)	<0.0001	1.19 (1.03–1.38)	0.017
Hypertension (yes vs. no)	1.07 (0.96–1.20)	0.21	1.05 (0.95–1.16)	0.34
Peptic ulcer (yes vs. no)	1.56 (1.40–1.73)	<0.0001	1.27 (1.14–1.43)	<0.0001
Smoking (yes vs. no)	1.42 (1.27–1.59)	<0.0001	1.18 (1.06–1,31)	0.002

CSC indicates central serous chorioretinopathy, NTD indicates New Taiwan Dollar, HR indicates hazard ratio, and CI indicates confidence interval. In the multivariable analysis, all the other variables in the table are included for adjustment.

**Table 4:** Subgroup analyses for the risk of depression among CSR patients, stratified by treatment.

Clinical outcome	With treatment *n* = 1297	Without treatment *n* = 24642
Depression		
** **Event	69	1492
** **Incidence density	100.06	86.27
** **Unadjusted HR	0.86 (0.68–1.10)	Reference
** **Adjusted HR	0.85 (0.67–1.08)	Reference

HR indicates hazard ratio. Adjusted HR was calculated after adjustment of age, gender, Charlson comorbidity index, insurance cost, urbanization level, types of steroid medications, hypertension, peptic ulcer, and smoking; the unit of incidence density: per 10000 year.

## Data Availability

Data are available from the National Health Insurance Research Database (NHIRD) published by Taiwan National Health Insurance (NHI) Bureau. The data utilized in this study cannot be made available in the manuscript, the supplemental files, or in a public repository due to the “Personal Information Protection Act” executed by Taiwan's government, starting from 2012. Requests for data can be sent as a formal proposal to the NHIRD (http://nhird.nhri.org.tw) or by email to wt.gro.irhn@drihn.

## Abbreviations

CSC: Central serous chorioretinopathy
NHIRD: National Health Insurance Research Database
ICD-9-CM codes: International Classification of Diseases, Ninth Revision, Clinical Modification codes
CCI: Charlson comorbidity index
HR: Hazard ratio
ATC code: Anatomic Therapeutic Chemical code
CI: Confidence interval.
